# Acute Focal Bacterial Nephritis and Bacteremia Due to Staphylococcus simulans Following an Upper Respiratory Infection in a Child: A Case Report

**DOI:** 10.7759/cureus.75191

**Published:** 2024-12-05

**Authors:** Akihisa Horigome, Hideko Uryu, Satoshi Takasago, Yukari Atsumi, Shinji Mochizuki

**Affiliations:** 1 Department of Pediatrics, Center Hospital of the National Center for Global Health and Medicine, Shinjuku, JPN; 2 Department of Cardiology, St. Luke’s International Hospital, Chūō, JPN; 3 Department of Pediatrics, Hyogo Prefectural Amagasaki General Medical Center, Amagasaki, JPN

**Keywords:** acute focal bacterial nephritis, bacteremia, contamination, hematogenous spread, staphylococcus simulans

## Abstract

Coagulase-negative *Staphylococcus* (CoNS) is a rare cause of UTIs in children and is often regarded as a contaminant in urine samples. We report a case of acute focal bacterial nephritis (AFBN) and bacteremia caused by *Staphylococcus simulans* following an upper respiratory infection in a pediatric patient. The patient, a four-year-old girl, presented with fever, cough, and a runny nose two days before being referred to our hospital due to persistent fever and poor oral intake. A multiplex nested PCR test of a nasopharyngeal swab sample detected human rhinovirus/enterovirus. Urinalysis showed no leukocyte esterase or nitrites, but revealed 1-4 white blood cells/high-power field and 1+ bacteria. Despite these findings, *S. simulans* was isolated from both urine and blood cultures. Contrast-enhanced abdominal CT revealed multifocal hypodense, wedge-shaped space-occupying lesions in both kidneys, characteristic of AFBN. This case underscores that *S. simulans* can cause severe UTIs and highlights the importance of considering CoNS, including *S. simulans*, as a potential UTI pathogen. The results of urine cultures should be interpreted in conjunction with other clinical findings, even when an alternative diagnosis for fever is present.

## Introduction

UTIs are among the most common bacterial infections in children, and prompt diagnosis and treatment are crucial to prevent acute complications and renal scarring [1]. *Escherichia coli *is the most common pathogen responsible for UTIs, with other frequent culprits including *Klebsiella*, *Proteus*, *Enterococcus*, and *Enterobacter* species [1]. Coagulase-negative *Staphylococcus* (CoNS) is a rare cause of UTIs in children and is typically regarded as a contaminant in urine samples, except for *Staphylococcus saprophyticus* in older children [1,2]. *Staphylococcus simulans*, a CoNS and a common animal pathogen, is rarely found on human skin and has been identified in only 0.2-2.1% of CoNS urinary isolates [3,4]. We report a case of acute focal bacterial nephritis (AFBN) and bacteremia caused by *S. simulans* following an upper respiratory infection in a pediatric patient.

## Case presentation

The patient was a four-year-old girl who presented with fever, cough, and a runny nose two days prior. She was referred to our hospital due to persistent fever ranging from 38.2 to 39.1°C and poor oral intake. Antipyretics were administered but proved ineffective. She had no prior history of UTI or fever of unknown origin and had not had close contact with any farm animals. Physical examination showed no signs of pneumonia, gastrointestinal disease, infective endocarditis, or Kawasaki disease. Blood tests revealed elevated levels of inflammatory markers, blood urea nitrogen, and uric acid (Table 1).

**Table 1:** Laboratory data on admission BUN, blood urea nitrogen; Cr, creatinine; CRP, C-reactive protein; HPF, high power field; LDH, lactate dehydrogenase; Lymph, lymphocytes; Mono, monocytes; Neutro, neutrophils; RBC, red blood cell; UA, uric acid; WBC, white blood cells

Blood test	Results	Reference range	Urine test	Results	Reference range
WBC	24.3 ×10^3^/μL	5.5-15.5 ×10^3^/μL	pH	5.5	4.5-7.5
Neutro	84.00%	39.7-71.2%	Specific gravity	1.032	1.005-1.030
Lymph	10.00%	21.9-50.3%	Protein	1+	-
Mono	6.00%	4.2-9.6%	Keton	3+	-
BUN	21.7 mg/dL	6.0-20.0 mg/dL	Nitrite	-	-
Cr	0.42 mg/dL	0.20-0.40 mg/dL	Leukocyte esterase	-	-
UA	8.7 mg/dL	2.6-7.0 mg/dL	WBC	1-4/HPF	0-5/HPF
CRP	14.8 mg/dL	0.00-0.14 mg/dL	RBC	<1/HPF	0-5/HPF
LDH	431 U/L	124-222 U/L	Bacteria	1+	-

**Figure 1:**
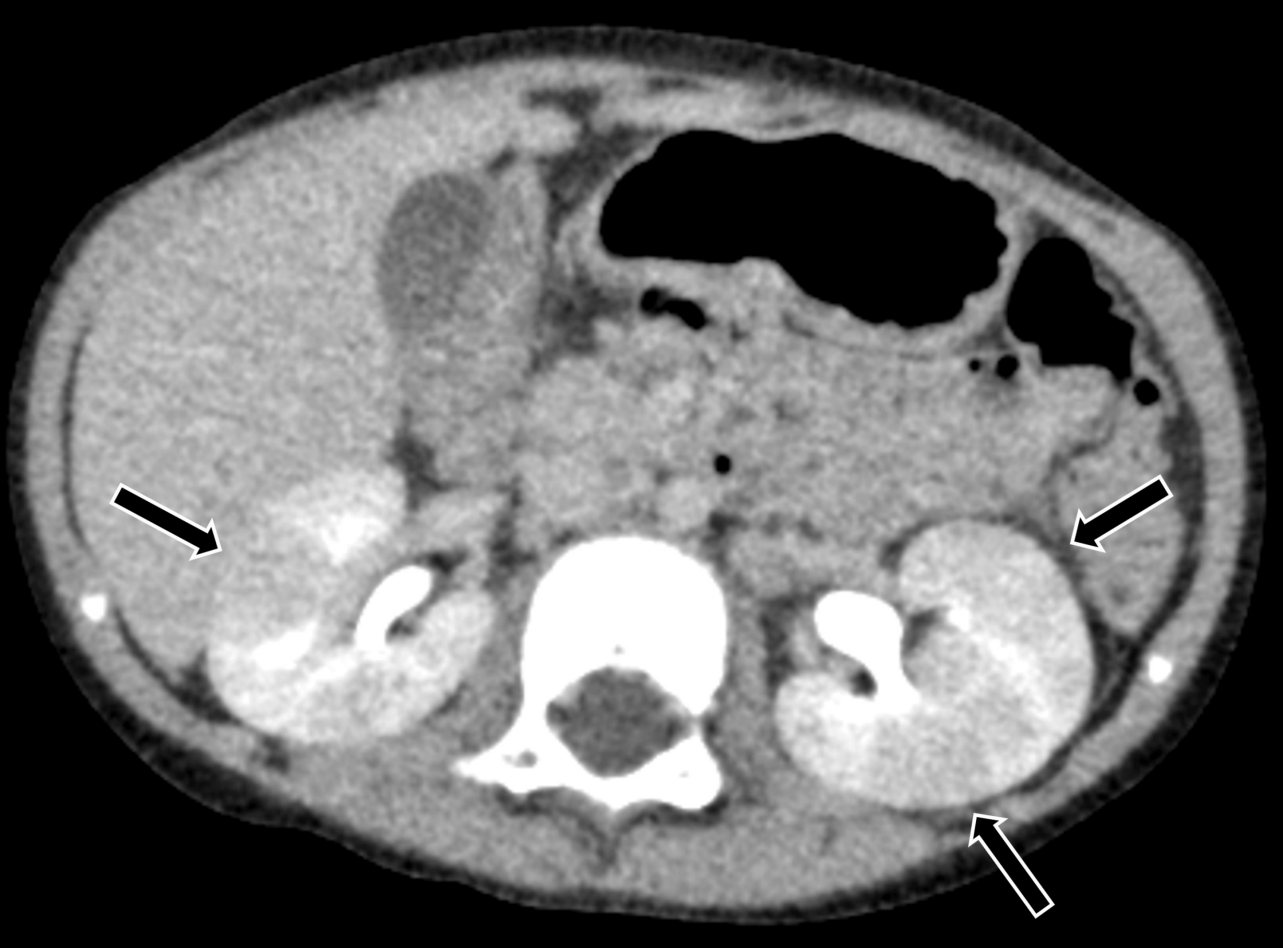
Contrast-enhanced abdominal CT scan showing multifocal hypodense, wedge-shaped space-occupying lesions in both kidneys (arrows)

A midstream clean-catch urine sample was obtained for urinalysis and urine culture. The urinalysis was negative for leukocyte esterase and nitrite tests, but it revealed 1+ bacteria and 1-4 white blood cells per high-power field. Gram-positive cocci were identified on urine Gram staining. Human rhinovirus/enterovirus was detected through a multiplex nested PCR test of a nasopharyngeal swab, and Group A Streptococcus (GAS) was identified by a rapid antigen test of a throat swab sample. The patient was treated with intravenous fluids for dehydration and ampicillin (200 mg/kg/day) for the GAS infection, starting on the day of admission. On the second day of admission, following the isolation of coagulase-negative staphylococci (CoNS) from the blood culture within 16 hours, the antibiotic was switched from ampicillin to vancomycin (40 mg/kg/day). The fever resolved by the third day of admission. The CoNS was identified as *S. simulans* by MALDI Biotyper, and the same bacteria were isolated from the urine culture at a concentration of more than 10,000,000 colony-forming units/mL. Contrast-enhanced abdominal CT revealed multifocal, hypodense, wedge-shaped, space-occupying lesions in both kidneys, characteristic of AFBN (Figure 1).

**Figure 2:**
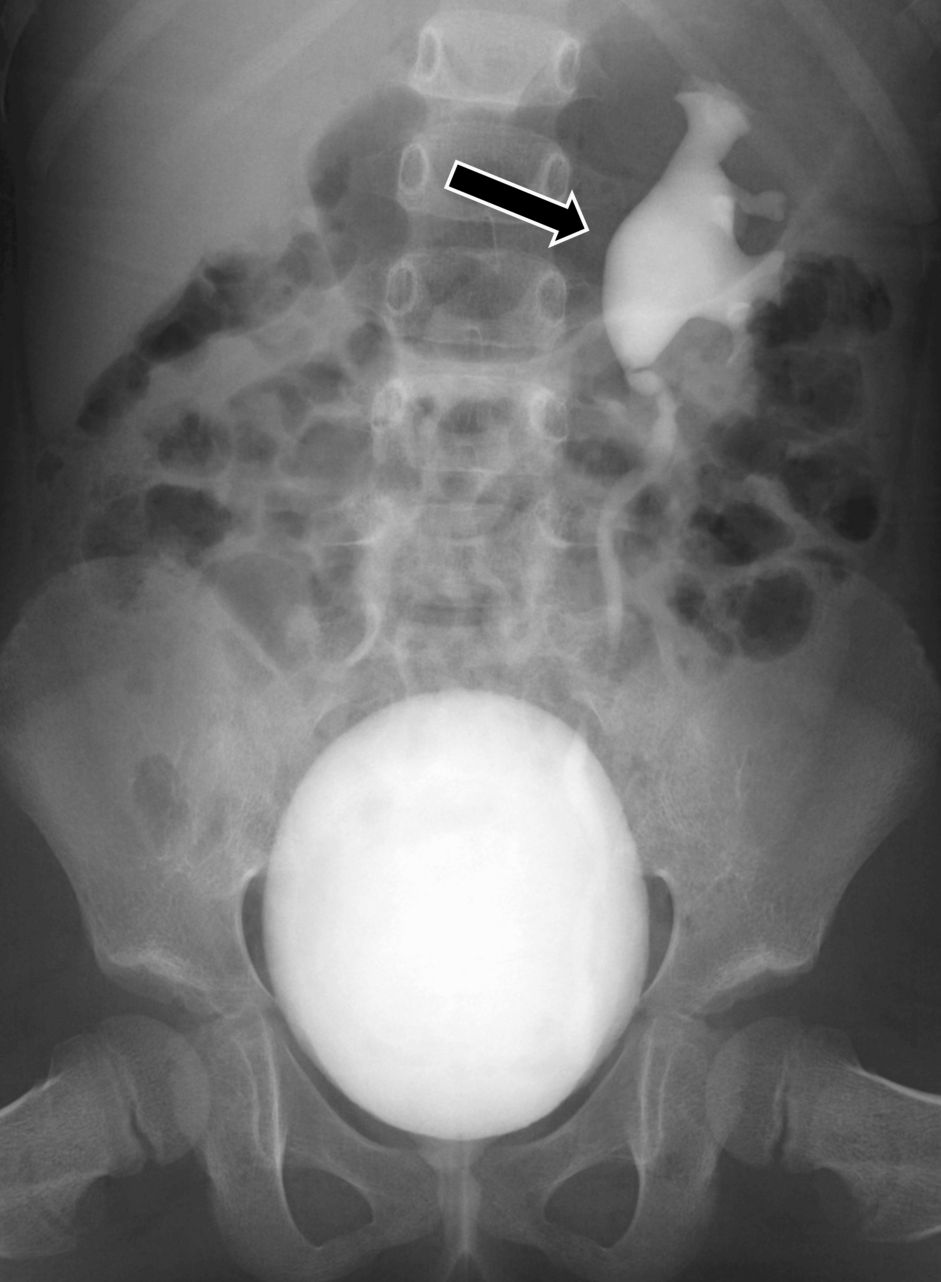
VCUG one month after onset showing grade 2 VUR on the left side (arrow) VCUG, voiding cystourethrography; VUR, vesicoureteral reflux

Transthoracic echocardiography showed normal results with no evidence of vegetation. The antibiotic was switched from vancomycin to cefazolin (150 mg/kg/day), to which the bacteria were susceptible (Table 2). After 14 days of intravenous antibiotic treatment, oral cephalexin (60 mg/kg/day) was prescribed for seven days. There was no recurrence of fever, and follow-up blood and urine cultures remained negative throughout the treatment course. Voiding cystourethrography (VCUG) was performed one month after treatment, and technetium-99m dimercaptosuccinic acid (99mTc-DMSA) renal scanning was done four months later. VCUG showed grade 2 vesicoureteral reflux (VUR) on the left side (Figure 2), and 99mTc-DMSA renal scanning revealed multiple areas of decreased radionuclide uptake in both kidneys, indicating renal scarring. Antibiotic prophylaxis with oral trimethoprim-sulfamethoxazole (TMP-SMX) (2 mg TMP/kg/day) was initiated. No recurrence of infection was reported during the three-year follow-up period with TMP-SMX prophylaxis.

**Table 2:** Antibiotic susceptibilities of isolated S. simulans The isolated* S. simulans *was susceptible to all tested antibiotics except ampicillin. MIC, minimal inhibitory concentration (μg/mL)

Antibiotic	Susceptibility	MIC
Ampicillin	Resistant	4
Ampicillin/sulbactam	Susceptible	<2
Cefazolin	Susceptible	<1
Minocycline	Susceptible	<1
Vancomycin	Susceptible	1
Teicoplanin	Susceptible	<1
Levofloxacin	Susceptible	<0.5
Sulfamethoxazole-trimethoprim	Susceptible	<0.5

## Discussion

*S. simulans* is a rare pathogen in children but can cause severe UTIs. As an opportunistic pathogen in farm animals, *S. simulans *is rarely found on human skin [5]. Although uncommon, *S. simulans* has been reported to cause soft tissue infections, endocarditis, bacteremia, osteomyelitis, prosthetic joint infections, and UTIs [6]. Previous studies suggest that patients with *S. simulans* infections often have close contact with farm animals [6,7]. However, our patient had no clear history of such exposure. Recent reports have described *S. simulans* infections in patients without contact with farm animals [2,8], suggesting that *S. simulans* may also be prevalent in urban environments, possibly from pets or stray animals. As cases of *S. simulans* infections increase, awareness of this pathogen is essential. To the best of our knowledge, this is the first report of concomitant AFBN and bacteremia caused by *S. simulans* in a child.

The growth of CoNS, including *S. simulans*, should not be dismissed as a contaminant in urine cultures from children, even when there is an alternative diagnosis, such as a fever or upper respiratory infection caused by rhinovirus/enterovirus, as seen in this case. A UTI should be suspected when no other source of fever is identified [1], although UTI can occur alongside respiratory viral infections [9]. Our patient had negative leukocyte esterase and urinary nitrite tests, as well as no pyuria, suggesting a lower likelihood of UTI. A two-step process for UTI screening in febrile young children involves initially screening for leukocyte esterase and nitrites using a noninvasive urine bag, followed by catheterization for those with positive results [10]. However, this approach may miss UTIs in cases like ours, where both tests were negative. The sensitivity of the leukocyte esterase test is only 79%, and nitrites are produced primarily by Gram-negative enteric bacteria, not Gram-positive organisms [1,11]. In this case, the mild respiratory symptoms compared to the blood test results prompted further investigation through urine and blood cultures, leading to the diagnosis. UTI should always be considered a potential cause of fever in children, especially when there is a mismatch between symptom severity and blood test results. In such cases, urine and blood cultures should be conducted to explore other diagnoses. CoNS, typically regarded as contaminants in urine cultures, can cause severe UTIs when present. Thus, *S. simulans* and other rare CoNS pathogens should be considered in the differential diagnosis, with urine culture results interpreted alongside clinical findings, even when an alternative diagnosis for fever is present.

The patient’s AFBN may have resulted from hematogenous spread rather than ascending infection from a lower UTI. AFBN is characterized by localized bacterial infection in the renal parenchyma, although its exact pathogenesis remains unclear [12]. It can result from an ascending infection, particularly in cases of lower UTIs, or from hematogenous spread, especially in younger children, following a respiratory tract infection [12]. Cases of AFBN with sterile urine cultures have also been reported [13]. In this case, urinalysis did not indicate a UTI, but both urine and blood cultures showed significant growth of *S. simulans*. Additionally, contrast-enhanced CT revealed multifocal hypodense lesions in both kidneys, and a 99mTc-DMSA renal scan showed renal scarring, while grade 2 VUR was present only on the left side, suggesting hematogenous spread. Although *S. simulans* can colonize human skin, our patient did not have a skin infection. Reports have also shown the presence of CoNS, including *S. simulans*, in human mucous membranes, such as the respiratory tract [14,15]. Therefore, we suspect that *S. simulans* may have been transmitted through damaged respiratory airways following a viral respiratory infection. It is important to consider routes of AFBN infection beyond the urinary tract.

## Conclusions

*S. simulans* can cause severe UTIs, and it is important to consider CoNS, including *S. simulans*, as potential UTI pathogens. Urine culture results should be interpreted alongside other clinical findings, even when an alternative diagnosis for a fever is present. Further studies are needed to identify risk factors for UTIs and the transmission routes of *S. simulans*.
